# Selenium Detection Technology in Water: A Review

**DOI:** 10.3390/molecules31040673

**Published:** 2026-02-15

**Authors:** Dan Wu, Weifang Bao, Fumin Xiong, Xingqian Ye

**Affiliations:** 1 Zhejiang Key Laboratory of Agri-Food Resources and High-Value Utilization, National-Local Joint Engineering Research Center of Intelligent Food Technology and Equipment, College of Biosystems and Food Science, Integrated Research Base of Southern Fruit and Vegetable Preservation Technology, Zhejiang International Scientific and Technological Cooperation Base of Health Food Manufacturing and Quality Control, Zhejiang University, Hangzhou 310058, China; 2 Zhejiang Luheng Water Quality Analysis and Intelligent Monitoring and Control Enterprise Research Institute, Zhejiang Lohand Environment Technology Co., Ltd., Hangzhou 310000, China

**Keywords:** selenium, water, detection, limit, health

## Abstract

Selenium (Se) is a trace element that is essential for the human body and has dual significant biological effects. The boundary between its ‘beneficial dosage’ and ‘toxic level’ is extremely narrow. Se is prone to accumulate in the body. Even if the concentration in drinking water is very low but consistently exceeds the limit, it may cause long-term health problems and pose risks and hazards to humans. Therefore, the detection of selenium is of great importance. The distribution and pollution of Se in water, the impact of Se on health and the limit requirements for Se in drinking water are introduced. The development of Se detection techniques is presented, including atomic spectrometry, spectrofluorometry, ultraviolet-visible spectrophotometry, inductively coupled plasma mass spectrometry, voltammetry, among others. Different analytical methods for selenium have their own characteristics and different applicability. It is necessary to establish a safety monitoring mechanism that primarily relies on laboratory-based instrumental analysis, supplemented by on-site rapid screening methods, to provide effective technical support for environmental Se analysis.

## 1. The Distribution and Pollution of Se in Water

Se is a widely distributed trace element in nature. Globally, the average Se concentration is 0.02 μg/L in freshwater, while it is typically below 0.08 μg/L in seawater [1,2]. Due to direct contact with rocks, groundwater exhibits a characteristically higher Se content than surface water [3]. The concentration of Se in surface water varies significantly with time and climatic conditions, the variation trend of Se concentration in surface water with time is flood season > mean flow season > dry season [4,5]. Dinh et al. found that the total concentrations of Se in drinking water in China have been reported to range widely between 0.017 and 46.0 μg/L; according to the different s Se content, drinking water can be classified into the following grades: Se deficiency < Se marginal < Se sufficient < Se rich < Se excessive [6].

Mines are the main source of selenium released into the environment. The production of Se is estimated to be approximately 2650 ± 150 tons per year [7]. Most Se exists in the form of associated elements in other elemental deposits. At present, the known independent Se deposits include El Dragon Se deposit, Pacajake Se deposit in Bolivia, and Yutangba Se deposit in Enshi, Hubei Province, China [8,9]. Mining activities represent the initial stage and critical process through which selenium is dispersed into the wider environment [10]. In China, the average content of Se in the Se mine can reach 3637.50 mg/kg, the maximum concentration can even reach 84,000 mg/kg in some mining areas; the Se concentration detected in surface water of Enshi was between 0.27 and 342.86 μg/L, and the Se concentration in drinking water was between 0.37 and 40.9 μg/L, which was higher than the global freshwater Se concentration of 0.2 μg/L [11]. The Se content in wastewater reported by other mines generally was 3–12,000 μg/L [3], with uranium mine wastewater at 1.6 mg/L and gold mine wastewater at 0.2 to 33 mg/L [12]. The Se coal mine groundwater is 3 to 330 μg/L [3]. The Se in the oil refinery wastewater is 15 to 75 μg/L [13,14].

## 2. The Impact of Se on Health and Its Limit Requirements in Drinking Water

Se has dual significant biological effects for human, its ‘beneficial dosage’ and ‘toxic level’ is extremely narrow. Both inadequate and excessive total intake disrupt homeostasis, leading to toxicity or deficiency-related pathologies, respectively, and resulting in various tissue dysfunctions [15]. The key properties of selenium—including its toxicity, mobility, bioavailability, and reactivity—are governed by its chemical speciation and oxidation state in aqueous environments. Se has four common oxidation states: −II (selenide), 0 (elemental Se), +IV (selenite), and +VI (selenate). Elemental Se is non-toxic because it is not easily dissolved and absorbed. Selenium compounds are classified into inorganic and organic species, with the inorganic forms exhibiting approximately 40 times greater toxicity than their organic counterparts. Selenium exhibits high water solubility in its +IV and +VI oxidation states, but low solubility in its elemental (Se^0^) and selenide (Se^−2^) forms. Organic Se has low toxicity, high bioactivity and high bioavailability. Selenomethionine, selenocysteine and other selenium-amino acid derivatives are available organic Se sources for humans and animals. Kieliszek and Bano [16] summarized mammalian selenoproteins and their functions in the body. According to Haug et al. [17], the major selenocompound differs by plant type: selenomethionine in cereal grains and legumes versus methylselenocysteine in Se-accumulator plants (e.g., garlic, onion, and broccoli).

Selenium deficiency is a known cause of Keshan disease and Kashin–Beck disease, and is also linked to an increased risk of Alzheimer’s disease [18,19,20]. Se supplementation can improve the body’s immunity and fertility, as well as anti-oxidant, anti-cancer, and anti-heavy metal [21,22,23,24,25,26]. However, the nutrient exhibits a narrow therapeutic window. Excessive intake leads to acute poisoning and chronic intoxication (selenosis), primarily caused by the sustained consumption of selenium-contaminated drinking water and crops cultivated in high-selenium soils [27]. The complex interplay between selenium status, human health, and disease is further modulated by the gut microbiota, a relationship comprehensively summarized by Wang et al. [28]. Several national health authorities, including those of Australia, Canada, China, New Zealand, South Korea, and the USA, set a common upper limit of 400 µg/day in their adult selenium intake guidelines.

Therefore, all countries have stipulated the maximum allowable content of Se in water. The limit of Se in drinking water in most countries or regions is 10–50 µg/L in Table 1, with 10 μg/L being the most common [29]. All countries around the world have recommended intake standards for selenium. In 2023, the Chinese Nutrition Society issued the Dietary Reference Intakes for Chinese Residents, which established the recommended dietary Se intakes for various population groups, as presented in Table 2 [30].

## 3. Selenium Detection Technology in Water

Selenium occurs in biological systems as multiple chemical species. Its bioavailability and toxicity are dictated by which species is present and at what concentration. Key considerations for selenium determination include accuracy, analyte concentration range, sample volume, sample matrix properties, and possible interfering substances. Current analytical approaches for selenium in water are largely founded upon the well-defined reactivity of Se(IV) species towards dedicated chemical compound. The determination of total Se is to convert various forms of Se into the same valence state to determine its total content. The methods for detecting selenium in water include hydride–atomic spectrometry, fluorescence spectrometry, ultraviolet spectrophotometry, chromatography, voltammetry, etc. Here, China is taken as an example, the standard detection methods for selenium in water were showed in Table 3.

In Table 3, atomic spectrometry and mass spectrometry require large-scale and expensive instruments, these methods need to be operated by professionals, have complex pretreatment (extraction, enrichment or separation steps) and high detection costs. However, their sensitivity (0.2–50 μg/L) does not have a significant advantage over spectrofluorometry or UV–Vis spectrophotometry (0.25–10 μg/L). Therefore, in general, just choose the spectrofluorometry or UV–Vis spectrophotometry method. At present, the instruments used in spectrofluorometry or UV–Vis spectrophotometry methods are quite widespread and affordable, making them more suitable for daily testing work. Especially in on-site rapid testing, since there are portable handheld colorimeters available for spectrofluorometry or UV–Vis spectrophotometry methods, the testing process is more convenient and faster. When the timeliness requirement for water quality detection is not strong and when detecting extremely trace amounts of selenium in water, atomic spectrometry and mass spectrometry may demonstrate some advantages by optimizing pretreatment methods. By comparing with Table 1 and Table 3, it can be seen that spectrofluorometry and atomic spectrometry can meet the detection requirements of drinking water in almost all countries. The detection sensitivity of other methods is relatively low, and it is necessary to conduct further in-depth research on sample enrichment and anti-interference in pretreatment.

### 3.1. Atomic Spectrometry

Atomic spectrometric methods, such as AAS and AFS, are commonly coupled with hydride generation (HG) technology. In this technique, selenium is converted into a gaseous hydride, effectively separating it from the aqueous sample matrix. AAS method has higher sensitivity compared with AFS in China standard method GB/T 5750.6-2023 (Table 3). Using a nanoscale nickel-aluminum layered double hydroxide as an effective solid-phase extraction (SPE) sorbent, Abdolmohammad-Zadeh et al. [35] achieved a detection limit of 10 ng/L for Se by continuous-flow HG-AAS. Ali et al. [36] employed ammonium pyrrolidine dithiocarbamate to complex with Se, forming a hydrophobic species. This complex was then extracted using a dispersing medium composed of Triton X-114 and the ionic liquid 1-butyl-3-methylimidazolium hexafluorophosphate. The Se hydrophobic complex was detected by graphite furnace AAS with a detection limit of 0.07 µg/L. Muslim et al. [37] formulated an extraction solvent from undecanoic acid and tetrabutylammonium hydroxide for application in air-assisted, solidified floating organic drop microextraction, coupled with HG-AAS for Se detection. A detection limit on the order of 0.07 µg/L was obtained. This method was more environmentally friendly and practical. Muhammet and Dilek [38] developed a new ultra-sensitive method for Se determination by combining a Pd-coated W-coil atom trap and hydride generation atomic absorption spectrometry (HG-AAS); its sensitivity was 38 times more than the conventional HG-AAS method. Tutar et al. [39] detected selenium in tap water samples by high performance liquid chromatography (HPLC) coupled with continuous flow HG (with online pre-reduction) and flame AAS. The limits of detection for Se(IV) and Se(VI) were 0.09 and 0.31 mg/kg, respectively, with corresponding quantification limits of 0.23 and 0.77 mg/kg. The Chinese National Standard GB/T 5750.6-2023 designates HG-AFS as the primary method for determining Se in water [31]. The direct determination of Se(IV) by HG-AFS is prone to interference owing to the analyte’s low concentration and the complex matrix of environmental samples. Thus, a separation or enrichment step is necessary prior to instrumental analysis. Lu et al. [40] determined Se (IV) in natural water by online polytetrafluoroethylene fiber-filled microcolumn preconcentration combined with HG-AFS. The method achieved a detection limit as low as 4 ng/L. Wang et al. [41] employed a method termed ultrasound-assisted back-extraction solidified floating organic drop microextraction (UA-BE-SFODME) with 1-undecanol. After chelation of Se(IV) with a water-soluble ligand, the analyte was extracted and then back-extracted into an aqueous solution under ultrasonication prior to HG-AFS determination, yielding a detection limit of 7.0 ng/L. It was seen that, no matter AAS method or AFS method, researchers mainly focused on pre-treatment optimization, focusing on selenium enrichment or extraction, in order to improve the detection sensitivity.

### 3.2. Spectrofluorometry

The 2,3-diaminonaphthalene fluorescence photometric method is common in spectrofluorometry; it is often a national standard method, such as China standard method GB/T 5750.6-2023 [31], 2,3-diaminonaphthalene reacts with Se(IV) in acidic solution to produce green fluorescent substances, which is extracted by cyclohexane to quantitatively determine the content of Se. The diamino naphthalene fluorescence method is a classical Se analysis method. It has high sensitivity, but the operation is cumbersome and time-consuming. Serra et al. [42] improved the determination method of selenium (IV) by taking advantage of the principle that selenite reacts with 2,3-diaminaphthalene (DAN), combined with SPE and automatic flow injection analysis techniques. A low detection limit of 1.7 μg/L was achieved. Muthu et al. [43] reported a rapid Se(0) detection method employing carbon nanodots (CNDs) generated from the Maillard reaction between glucose and glycine; the CNDs were used as fluorescent probes, with significant sensitivity and selectivity, and their detection limit was 0.381 mmol/L. A fluorescent probe based on microwave-synthesized Sn-doped carbon quantum dots was developed by Thakur et al. for Se(IV) detection. The method utilizes the specific fluorescence quenching caused by the Se-Sn complex formation, demonstrates selectivity in the presence of interfering ions, and achieves a detection limit of 0.011 μg/L [44]. Aimaitiniyazi et al. [45] proposed a visual detection method with DAN as fluorescent ligand and 9-anthrene ethanol as the fluorescent reagent, enabling a sensitive detection limit of 1.6 µg/L for Se. The fluorescence-based visual detection of Se was successfully realized on a portable smartphone platform. The emergence of the fluorescent probe to improve the sensitivity of spectrofluorometry will be the direction of future development and research.

### 3.3. Ultraviolet-Visible Spectrophotometry

The ultraviolet-visible spectrophotometry is simple and easy to operate, but it is limited by insufficient sensitivity and selectivity, particularly for detecting trace levels of selenium in environmental samples. Therefore, it usually combines with extraction and concentration for determination. The reaction of 3,3′-diaminobenzidine (DAB) and Se (IV) is a traditional method, whose chemical reaction is as follows:

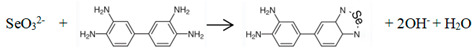
Selenite 3,3′-Diaminobenzidine 3,4-diaminophenyl piazaselenol(1)

**Figure 1:**
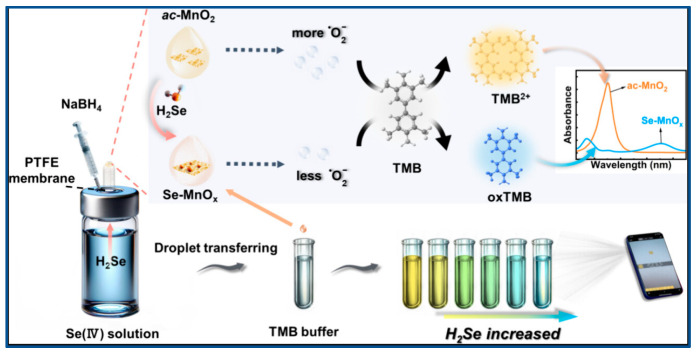
Colorimetric reaction for Se(IV) determination based on H_2_Se, ac-MnO_2_ and TMB [51].

After the water sample was digested with mixed acid, the selenium element inside was converted into Se (IV), it reacted with DAB to yield a yellow product, which was subsequently extracted into toluene, and then measured at 420 nm by chromometer [32]. The functional group of 3,3′-diaminobenzidine reacting with Se is the o-diamine, and its semi-molecule o-phenylenediamine has the functional group that can react with Se. Chen et al. [46] analyzed selenium in water of hot spring by ultraviolet-visible spectrophotometry with o-phenylenediamine as color reagent. Its detection wavelength was 333 nm, the linear relationship was from 0.05 to 0.40 μg/mL. Cloud point extraction (CPE) is a surfactant-based separation technique characterized by temperature-induced phase separation, falling under the category of liquid–liquid extraction. Sounderajan et al. [47] reported a method for determining Se(IV) and Se(VI) in water using cloud point extraction (CPE), which utilized the formation of a Se-DAB complex with the surfactant Triton X-114, achieving a detection limit of 0.0025 μg/L. In a separate study, Agrawal et al. [48] determined Se(IV) based on the formation of an ion-association complex, with a reported detection limit of 10 ng/mL. Alternatively, Nayanova et al. [49] employed different mechanisms for speciation: Se(IV) was quantified via its oxidation of methylene blue, while Se(VI) was detected through ion-pair formation with a specific reagent, yielding detection limits of 1 μg/L and 0.8 μg/L, respectively. Moving towards more integrated detection platforms, Xiong et al. [50] developed a multimodal approach. They first separated Se(IV) using a SPE with thiol cotton. Subsequently, leveraging the catalytic effect of selenium on the iodine-starch reaction (which produces a blue color), they established three detection modes: spectrophotometric, smartphone app-based colorimetric, and visual (naked-eye) colorimetric analysis. The methods exhibited varying performance: the spectrophotometric mode showed a linear range of 0.01–8.0 µg/mL and a detection limit of 0.0047 µg/mL; the smartphone-based mode was linear from 1.0 to 6.0 µg/mL with a limit of 0.05 µg/mL; and the visual mode could reliably distinguish Se levels as low as 0.1 µg/mL. Wang et al. [51] reduced Se(IV) in samples into volatile selenium hydride (H_2_Se) by chemical reaction, H_2_Se oxidized with ac-MnO_2_ and the Se-MnOx was produced (Figure 1). A detection platform was established based on the selenium-catalyzed oxidation of 3,3′,5,5′-tetramethylbenzidine (TMB) to a blue chromogen. The resulting colorimetric signal varied linearly with Se(IV) concentration, enabling visual detection across a concentration range of 10–600 μg/L with a limit of detection of 1.8 μg/L. How to make visible spectrophotometry more convenient, rapid, highly sensitive and easily directly judged by the human eye will be an important research direction for ultraviolet-visible spectrophotometry.

### 3.4. Inductively Coupled Plasma Mass Spectrometry

Inductively coupled plasma mass spectrometry (ICP-MS) is commonly coupled with chromatographic separation techniques, such as gas chromatography (GC) and high-performance liquid chromatography (HPLC). GC can directly quantify volatile organoselenium compounds. In contrast, the analysis of inorganic selenium species typically requires their prior derivatization into volatile forms, such as selenium hydrides (e.g., H_2_Se) or alkylated derivatives. Species-specific isotope dilution are excellent methods for accurately quantifying element speciation. A highly sensitive method for selenium speciation was reported by Breuninger et al. [52], based on species-specific isotope dilution GC-ICP-MS. The low detection limits were achieved by maximizing the pre-concentration factor through optimized operational parameters, including increased sample and injection volumes as well as tailored injection protocols for different selenium species. The detection limit of this method reached 0.9–3.1 ng/L. HPLC coupled with ICP-MS is a standard technique for separating and detecting non-volatile selenium species, such as Se(IV) and Se(VI), in aqueous samples. For instance, Martínez-Bravo et al. [53] employed anion-exchange HPLC-ICP-MS for the determination of selenium species, achieving detection limits of 1.2 μg/L for Se(IV) and 1.4 μg/L for Se(VI). This method also enabled the simultaneous quantification of arsenic, selenium, and chromium(VI). In another approach, Luo et al. [54] integrated HPLC separation with photochemical vapor generation (PVG)-ICP-MS. After separation, the Se(IV) and Se(VI) fractions were mixed with MIL-125-NH_2_ nanoparticles and formic acid in a PVG reactor. Under ultraviolet irradiation, volatile selenium species were generated and subsequently determined by ICP-MS. This method achieved low detection limits of 0.8 ng/mL for both species, with relative standard deviations of 4.6% and 3.7%, respectively. Song et al. [55] analyzed the nano-Se (nSe) captured by polyvinylidene fluoride and nylon microporous filtration membranes by ICP-MS, its retention rate was more than 91.0 ± 0.87%. Ionic selenium species (selenite and selenate) in the filtrate were then quantified by HPLC-ICP-MS, enabling the distinct analysis of particulate and ionic Se fractions. Method validation showed recoveries of 70.2–85.8% for nSe (0.2 µg/L spike) and 83.6–101% for co-existing Se(IV)/Se(VI) (0.55 µg/L each). This work highlights the key strength of ICP-MS: its ability to quantify selenium across diverse physicochemical forms within an integrated analytical workflow. In contrast, other methods usually convert different forms of selenium into a single form of inorganic selenium for determination through pre-treating (especially digesting), mainly to measure the total selenium content. For instance, the ICP-MS method in China standard GB/T 5750.6-2023 directly states that this method allows for the simultaneous determination of five selenium species with the following detection limits: selenocysteine, 1.0 μg/L; methylselenocysteine, 1.4 μg/L; selenite, 1.0 μg/L; selenomethionine, 2.0 μg/L; and selenate, 1.0 μg/L [31]. It is therefore particularly well-suited for assessing the speciation of selenium in aquatic environments.

### 3.5. Voltammetry

In voltammetry, the method involves first concentrating ions on the electrode surface by electrodeposition and then applying a reverse voltage sweep to strip them, which generates the analytical signal [56]. Voltammetry typically uses stripping analysis with a pre-enrichment step; it can achieve lower detection limit. Voltammetry includes adsorptive cathodic stripping voltammetry and anodic stripping voltammetry. The working electrodes include glassy carbon electrode, hanging mercury electrode, mercury plating electrode and so on. Recently, electrode surfaces have been engineered with diverse functional materials to improve key detection parameters such as sensitivity, selectivity, and stability. The materials included carbon-based nanomaterials, noble metals and conductive polymers [57,58,59,60].

**Figure 2:**
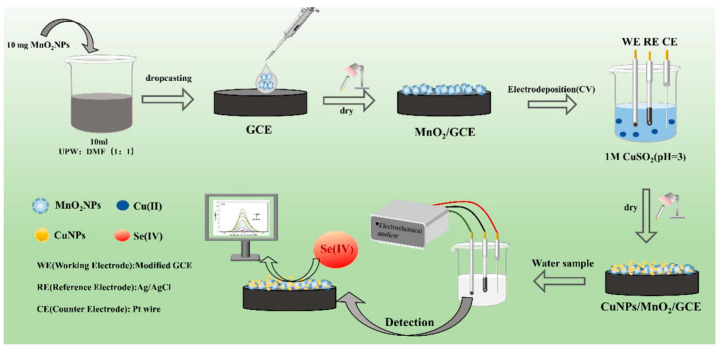
The preparation of the CuNPs/MnO_2_/GCE [68].

Ashournia and Aliakbar [61] found that Se(IV) and I^−^ can react quickly and completely to produce Se-I_2_ under acid catalysis. They used bovine albumin as the medium for adsorption accumulation of Se-I_2_ on a thin mercury film electrode, and the adsorbed Se-I_2_ was stripped in HCl solution by differential pulse cathodic potential scanning. The enhancement effect of bovine albumin on Se-I_2_ adsorption directly leads to the increased sensitivity of the method. Ashournia and Aliakbar [62] developed a thin mercury electrode based on the principle that o-phenylenediamine reacts with Se (IV) in acidic solution to produce 5-nitropiazselenol, which has a high tendency of self-accumulation on it. Se was determined by the cathodic stripping of adsorbed 5-nitropiazselenol in HCl. Separately, Ramadan et al. [63] employed a vitamin E/Nafion-modified gold electrode for the pulse anodic stripping voltammetry (DPASVA) determination of Se(IV). The sensitivity was 200 times more than the bare gold electrode. Ramadan, Mandil and Ozoun [64] studied the DPASVA at a methylene blue-nafion modified gold electrode. The sensitivity was 5-fold higher than that achieved with a vitamin E/Nafion-modified gold electrode, and 1000-fold higher than that of a bare gold electrode. Ramadan et al. [65] studied the DPASVA using a gold electrode modified with DAB; its sensitivity was more than 20000 times than the bare gold electrode. Ramadan et al. [66] developed a DPASVA method using a gold electrode modified with a composite of o-phenylenediamine and nafion. The sensitivity was 2000 times more than the bare gold electrode. Tan et al. [67] fabricated a glassy carbon electrode (GCE) modified with gold nanocages/fluorinated graphene nanocomposite for selenium determination via square wave anodic stripping voltammetry, achieving a detection limit of 0.27 μg/L. In a separate study, Yue et al. [68] developed a sensor by sequentially immobilizing manganese dioxide and electrodepositing copper nanoparticles onto a GCE (Figure 2). This design leveraged the synergistic effects of the nanocomposite to enhance Se(IV) adsorption and electron transfer, resulting in a detection limit of 3.7 μg/L within a linear range of 0.01–1 mg/L. Voltammetry offers significant advantages, including high sensitivity, short analysis time, a compact footprint, and ease of operation, making it an attractive analytical technique. It has attracted much attention in the in situ rapid detection of selenium ions. However, it is still necessary to design and synthesize suitable working electrode modification materials to make them have better stability, anti-interference ability, high detection sensitivity and lower detection limit, so as to achieve in situ rapid detection of water samples in the field.

### 3.6. Other Methods

Beyond the conventional techniques discussed, a variety of alternative methods have been developed for selenium determination. A common challenge in such analyses is the need for effective separation and preconcentration, particularly in matrices containing high concentrations of competing ions like sulfate (SO4^2−^). Addressing this, Nakakubo et al. [69] employed dithiocarbamate-modified cellulose (DMC) for the selective extraction and preconcentration of selenium, followed by quantitative analysis using portable liquid electrode plasma–optical emission spectrometry. The DMC adsorbent exhibited remarkable selectivity for Se(IV), enabling quantitative recovery even in the presence of high concentrations of sulfate ions (SO4^2−^). In a different approach leveraging bio-recognition, Yang et al. [70] developed an indirect competitive enzyme-linked immunosorbent assay for selenium. This immunoassay, based on hapten modification and bioconjugates, exhibited a linear range of 17–207 pmol/mL and a detection limit of 3.9 pmol/mL. Validation using four different selenium compounds in water samples yielded satisfactory recoveries (80–108%) with coefficients of variation (CVs) between 2.1% and 11%. The continuous advancement of analytical chemistry and instrumental science promises the further development of increasingly sophisticated, sensitive, and selective techniques for selenium detection.

## 4. Conclusions

With the expansion of industrial and economic activities, Se pollution is inevitable, and the analysis technology of Se becomes particularly important. The ICP-MS method and electrochemical methods can analyze different forms of inorganic and organic selenium; other methods can only determine a single form of selenium. But electrochemical methods are currently mainly focused on the detection of Se(IV). Therefore, the ICP method has a good advantage in the multi-form analysis of selenium in environmental samples. The instruments required for spectrofluorometry and ultraviolet-visible spectrophotometry are relatively economical and practical, have a high popularity rate, and are easy to operate. The instruments used in atomic spectrometry and ICP-MS are expensive, bulky, complex to operate and require more professional technicians. Spectrofluorometry has higher sensitivity and selectivity compared with ultraviolet-visible spectrophotometry, and its sensitivity can catch up with atomic spectrometry. By synthesizing fluorescent probes and adding fluorescent reagents, its anti-interference ability can be improved. This method holds promise as a key direction for future advancements in selenium determination. Voltammetry is a powerful tool for in situ rapid detection, with its sensitivity and selectivity being primarily governed by the electrochemical properties of the sensor or electrode material.

Selenium compounds in the natural environment vary significantly at different times and spaces. Therefore, it is necessary to select the detection method based on the actual situation, such as the sensitivity requirements for detection, whether multi-form selenium analysis is needed, and the requirements for detection timeliness, etc. Various detection methods complement each other, establishing a safety monitoring mechanism with large-scale instrument analysis as the main body and on-site rapid screening detection methods as a supplement, providing effective technical support for the selenium pollution monitoring under actual environmental conditions.

## Figures and Tables

**Table 1:** Maximum acceptable concentration of Se in drinking water as stipulated by various agencies worldwide [29].

Countries or Organizations or Agencies	Maximum Acceptable Concentration (µg/L)
UNICEF, Quebec, Saskatchewan, New Brunswick, Ontario, British Columbia, Oklahoma, Peru, Oceania Australia, Papua New Guinea, Fiji, Panama, Bosnia and Herzegovina, Serbia, Costa Rica, United Kingdom, Germany, Iceland, Norway, Russia, Belarus, Turkey, France, Chile, Bolivia, Argentina, Cambodia, China, Taiwan, Cuba, Israel, Colombia, Japan, India, Bangladesh, Malaysia, Thailand, Yemen, Mozambique, Morocco, Rwanda, Egypt, Ecuador, Ethiopia, East African Community, Uganda, Tanzania	10
Jordan	15
European Union, Switzerland,	20
California Environmental Protection Agency	30
WHO, New Zealand, Brazil, Mexico, Abu Dhabi in United Arab Emirates, Singapore, Saudi Arabia	40
Health Canada, US EPA, California, Ukraine, Dominican Republic, South Africa,	50

**Table 2:** Dietary selenium reference intakes of Chinese residents [30].

Age	Average Demand (µg/Day)	Recommended Intake (µg/Day)	The maximum Tolerable Intake (µg/Day)
0~	–	15	55
0.5~	–	20	80
1~	20	25	80
4~	25	30	120
7~	30	40	150
9~	40	45	200
12~	50	60	300
15~	50	60	350
18~	50	60	400
Pregnancy	54	65	400
Lactation period	65	78	400

**Table 3:** National and local standard detection methods for selenium in water in China.

Method	Name	Range μg/L	Standard Number
Spectrofluorometry	2,3-Diaminonaphthalene Fluorometric Method	>0.25	GB/T 5750.6-2023 [31]
UV–Vis spectrophotometry	3,3′-Diaminobenzidine Spectrophotometric Method	>8.00	HJ 811-2016 [32]
	Iron(II)-o-Phenanthroline Indirect Spectrophotometric Method	10–200	SL/T 272-2001 [33]
Atomic spectrometry (AS)	Hydride Generation (HG)-Atomic Fluorescence Spectrometry (AFS)	0.50–20.00	GB/T 5750.6-2023 [31]
	Hydride Generation (HG)-Atomic Absorption Spectrophotometry (AAS)	>0.20	
Inductively coupled plasma (ICP) mass spectrometry	ICP Mass Spectrometry	>1.64	HJ 700-2014 [34]
	Liquid Chromatography-ICP Mass Spectrometry	>1.00	GB/T5750.6-2023 [31]
	ICP Optical Emission Spectrometry	>50.00	

## Data Availability

No new data were created or analyzed in this study. Data sharing is not applicable to this article.

## Abbreviations

AAS	Atomic absorption spectrometry
AFS	Atomic fluorescence spectrometry
AS	Atomic spectrometry
CNDs	Carbon nanodots
CPE	Cloud point extraction
DAB	3,3′-diaminobenzidine
DAN	2,3-diaminonaphthalene
DMC	Dithiocarbamate-modified cellulose
DPASVA	Differential pulse anodic stripping voltammetry
GC	Gas chromatography
GCE	Glassy carbon electrode
H2Se	Selenium hydride
HG	Hydride generation
HG-AAS	Hydride generation atomic absorption spectrometry
HG-AFS	Hydride generation atomic fluorescence spectrometry
HPLC	High performance liquid chromatography
ICP	Inductively coupled plasma
ICP-MS	Inductively coupled plasma mass spectrometry
nSe	Nano-Se
TMB	3,3′,5,5′-tetramethylbenzidine
Se	Selenium
SPE	Solid-phase extraction
WHO	World Health Organisation
UNICEF	United Nations Children’s Fund
US EPA	United States Environmental Protection Agency
